# Adapting a National Framework to Inform Curricular Redesign Focused on Enhancing Student Clinical Competency

**DOI:** 10.3390/pharmacy9020089

**Published:** 2021-04-22

**Authors:** Andrew Bzowyckyj, Bridget Bradley, Pauline Cawley, Brandon Nuziale, Sarah White

**Affiliations:** School of Pharmacy, Pacific University Oregon, Hillsboro, OR 97123, USA; bridget.bradley@pacificu.edu (B.B.); cawleyp@pacificu.edu (P.C.); brandon.nuziale@pacificu.edu (B.N.); sarah.white@pacificu.edu (S.W.)

**Keywords:** pharmacy education, curriculum redesign, framework, clinical competency, APPE readiness, practice readiness

## Abstract

Doctor of Pharmacy (PharmD) programs continually engage in curricular redesign to ensure practice readiness of graduates. With ever-increasing demands on clinical competency and curricular time, it is important to be intentional when determining curricular priorities and prioritize contemporary pharmacist practice. This paper describes how to adapt a national framework for pharmacotherapy curricula to emphasize the pharmacist’s role within a given topic area in order to facilitate conversations about allotting curricular time during a curricular redesign. Customized Tier descriptions based on various factors expected of student pharmacists during Advanced Pharmacy Practice Experiences (APPEs) were developed (e.g., relative autonomy of the pharmacist in managing the topic, emphasis on licensing exams, frequency with which students can expect to encounter the topic at school-specific experiential placements, and condition-specific information). Topics were also reprioritized to address regional variations in practice and ideologies. Customizing a national framework to determine program-specific considerations for prioritizing topics within the pre-APPE curriculum can help faculty and students alike maintain focus on highly critical and foundational concepts, while also making sure not to completely disregard topics of lower priority. We have proposed such a framework for programs to utilize when facilitating conversations surrounding curricular reforms and topic prioritization.

## 1. Introduction

A multitude of Doctor of Pharmacy (PharmD) programs across the United States are engaging in curricular redesigns as a result of several factors, including advancements in pharmaceutical research and development, increased reliance on medications for managing chronic conditions, continual evolution of pharmacy practice standards and scope of practice, and the corresponding implementation of new accreditation standards from the Accreditation Council for Pharmacy Education (ACPE) in 2016, among numerous other factors [1,2]. A large emphasis of this initiative is a focus on “practice ready” graduates, with a corresponding emphasis on “APPE (Advanced Pharmacy Practice Experiences) ready” students, but a clear definition for either term has been difficult to articulate [2,3,4,5]. The typical PharmD curriculum entails an initial 2–3 years of didactic classroom instruction, pharmacist skills labs, and authentic practice experiences during Introductory Pharmacy Practice Experiences (IPPEs). These years are followed by 1–2 additional years of experiential education in authentic practice settings (i.e., APPEs). Although the steady increase in medication options to treat patients’ medical conditions and expansions of the pharmacist’s scope of practice are positive trends, the amount of time available in the PharmD curriculum is finite. Therefore, in order to assure clinical competency of students transitioning from the didactic to the experiential curriculum, content must be prioritized to align with contemporary pharmacist practice.

The existing literature provides several examples of strategies to evaluate curricular content and make critical decisions regarding content prioritization. Within pharmacy education, Longyhore, Dixon and Noel propose using a modified Ebel grid for setting curricular boundaries in pharmacy school by evaluating topics on a scale from “need to know” to “nice to know”, taking into consideration a topic’s criticality and relevance [6]. Outside of pharmacy education, other models proposed for evaluating curricular content include shifting the emphasis of course content from acquiring textbook knowledge to developing scientific thinking skills through the use of journal articles, the use of concept mapping when performing quality improvement on individual courses and entire curricula, and the use of the “understanding by design” framework for purposeful planning of curricula [7,8,9]. Despite these helpful resources, this process can still be difficult to operationalize beyond the individual course level at an institution because they all rely on the assumption that faculty generally agree on what is considered important.

Whenever making decisions about the curriculum, either during a complete redesign or a routine self-assessment for quality improvement, it is helpful to have external references to provide a “standard” against which to benchmark. The ACPE Standards provide that resource for the overall PharmD, while also affording programs a certain degree of flexibility to meet the standards [2]. To provide guidance regarding the pharmacotherapy curriculum specifically, the American College of Clinical Pharmacy (ACCP) publishes and periodically updates their Pharmacotherapy Didactic Curriculum Toolkit, with the most recent update occurring in 2019 [10]. In this document, recommendations are provided on how to prioritize 302 various pharmacotherapy topics distributed amongst three separate tiers, with an emphasis on practice competence rather than topic coverage. The factors deciding which condition belongs in which Tier include the global burden of each condition, pharmacotherapy available for the condition, the pharmacist’s role in optimizing pharmacotherapy, and the overall complexity of pharmacotherapy [10]. Descriptions of the three different Tiers with topic exemplars are featured in Table 1. The toolkit recommends Tier 1 topics be especially emphasized in pharmacy curricula, Tier 2 topics be introduced to students through a mix of required and elective coursework, and acknowledge that there is likely not enough time to adequately introduce Tier 3 topics. A complete list of all topics included within the ACCP Pharmacotherapy Didactic Curriculum Toolkit can be found at the following link: https://www.accp.com/docs/positions/Other_Documents_of_Interest/Flannery_jac5.1178.pdf (Accessed on 21 April 2021).

Although the design of the toolkit is helpful for discerning between high and low priority topics to be emphasized within PharmD curricula, subsequent application to inform the appropriate breadth and depth of content coverage necessary to achieve clinical competence for each individual topic is difficult. This is especially true for programs that integrate the foundational and clinical sciences throughout their coursework—how might these tier descriptions help inform faculty within the pharmaceutical sciences? A modified Ebel grid as proposed by Longyhore and colleagues can help, but incorporating an additional level of detail beyond what the ACCP Pharmacotherapy Toolkit provides can ensure these decisions are more consistently applied across the wide range of topics [6]. The aim of this paper is to share a framework for prioritizing curricular content to help other institutions facilitate conversations among faculty about streamlining the pharmacotherapy curriculum to help enhance clinical competency upon graduation.

## 2. Materials and Methods

Pacific University School of Pharmacy (SoP), a 3-year PharmD program, initiated the process of revising the PharmD curriculum in 2017 for implementation in Fall 2021, partly in response to the dramatic changes in contemporary pharmacy practice since the curriculum was originally adopted in 2006. The most significant change includes a shift from a modified 2-week block curriculum to an integrated, semester-based one. This shift towards integrated coursework is helping the faculty break through the silos that traditionally exist in pharmacy curricula, especially those related to organ system/specialty area and discipline. The steering group responsible for overseeing the curricular revision consists of eleven faculty and staff members (which includes representatives from the curriculum committee): two co-chairs, four pharmacy practice representatives, three pharmaceutical science representatives, the Director of Curricular Operations, and the Assistant Dean for Educational Outcomes.

The ACCP Pharmacotherapy Didactic Curriculum Toolkit was certainly helpful for framing the initial conversations during the process by serving as a national standard for what to prioritize and provided a framework to consider when prioritizing topics. Although the methods regarding topic prioritization were appropriate, institution-specific customization was warranted to account for state/region-specific scope of practice expectations, local prevalence of certain conditions, and populations served at available experiential settings for students, amongst other factors. Therefore, a small number of topics were reprioritized based on faculty feedback to accommodate these variations. During these conversations, it was generally understood by all that Tier 1 topics are high priority and Tier 3 topics likely would not be covered in our curriculum, but the 133 topics designated as Tier 2 were more difficult to conceptualize because they fell somewhere in between the two extremes. In addition, it was difficult for many to conceptualize how each topic’s prioritization translated into teaching time and content coverage, recommendations that the ACCP Toolkit intentionally avoided by design. With definitions geared towards practice expectations after graduation, an adaptation of the framework was needed.

The importance of developing clear guidance to faculty for what each final Tier placement would mean for individual topics was identified to be essential. The most common assumption was that higher priority topics automatically warranted more time, which was not inherently true. A topic of higher priority may actually need less time to achieve the desired level of competence, while a topic of lower priority may actually take more time, even if the expected level of competence may be lesser. Therefore, the steering group brainstormed the various factors, including the knowledge, skills, and abilities expected of student pharmacists during Advanced Pharmacy Practice Experiences (APPEs) through the lens of the various topics/conditions within each tier. Factors that were identified during the initial brainstorm include the relative autonomy of the pharmacist in managing the topic/condition, the emphasis of the topic/condition on licensing exams, the frequency with which students can expect to encounter the topic/condition at our school-specific experiential placements, and condition-specific information including treatment guidelines, goals of therapy, and medication-specific knowledge (especially important for faculty in the pharmaceutical sciences). The steering group used these criteria to develop program-specific descriptions for each tier.

To address the need for better differentiation between the 133 different Tier 2 topics, each member of the steering group was invited to participate in an anonymous survey to re-prioritize each topic with three options for each Tier 2 topic—“Higher priority (Tier 2A)”, “Lower Priority (Tier 2B)”, and “Not a priority (move to Tier 3)”. After approximately one week, results were tabulated. Items with ≥50% agreement for topic prioritization were considered as having achieved consensus and were declared final. Items with 50% agreement for multiple tiers or not achieving 50% agreement were reconciled through discussion among the steering group and group consensus. A similar process was utilized for differentiating between the Tier 1 topics to further stratify topics (if necessary), and although Tier 1A topics were identified as having the highest priority, this level of stratification did not result in any meaningful degree of differentiation in criteria for the purposes of the curricular redesign.

Lastly, the original ACCP Toolkit is designed around a combination of major organ systems (e.g., cardiovascular, endocrine), specialty practice areas (e.g., toxicology), and individual populations (e.g., OB/GYN, geriatrics). Although this approach works well when thinking as a clinical practitioner, it does not necessarily work as well when thinking about a student pharmacist learning about how to use these medications for the first time (especially in an integrated curriculum). For example, although “psoriasis” is appropriately categorized as a dermatologic condition and “rheumatoid arthritis” is appropriately categorized as a musculoskeletal condition, the treatments are similar so these topics make sense to be co-located near each other.

Once the program-specific tier descriptions were developed and all topics were prioritized by the steering group, the final results were disseminated to the entire faculty and staff for a two-week comment period. Comments related to the program-specific tier descriptions, the prioritization of individual topics, and the categorization of individual topics were solicited via email and a series of five one-hour town hall forums. All comments received were subsequently considered by the steering group and addressed as appropriate prior to declaring the documents final.

## 3. Results

### 3.1. Program-Specific Tier Descriptions

The final program-specific descriptions for each Tier are provided in Table 2. To assist in comparisons between the different Tiers, similar terminology was used for each Tier, with the relevant differences noted using bold text.

### 3.2. Prioritization and Categorization of Individual Topics

Over the course of the two-week commentary period, the steering group received 44 unique comments on topic prioritization (e.g., which tier does a topic fall under), an additional 59 unique comments related to topic categorization (e.g., which topic falls under which category), and no comments or suggestions for the program-specific Tier descriptions. All comments were reconciled by the steering group with a response formulated for each comment, regardless of outcome (accepted vs. rejected). The original ACCP Pharmacotherapy Toolkit identified 94 Tier 1, 133 Tier 2, and 75 Tier 3 topics [10]. The final program-specific topic area prioritization resulted in 70 Tier 1A, 29 Tier 1B, 71 Tier 2A, 61 Tier 2B, and 78 Tier 3 topics. The net gain of 7 topics is a result of some topics in the ACCP Pharmacotherapy toolkit being split apart to accommodate placement in multiple priority areas according to recommendations from individual faculty members. For example, “peptic ulcer disease (including stress-related mucosal injury, gastrointestinal bleeding” was separated into two discrete topics: “peptic ulcer disease (gastrointestinal bleeding)” and “stress ulcer prophylaxis”.

## 4. Discussion

Often, in an attempt to have a comprehensive curriculum that attempts to emphasize all topics, students risk becoming overloaded and unable to apply the material within a broader clinical context. Rather, the end result is a brain full of unintegrated knowledge and facts. In order to ensure clinical competency, a methodical approach to topic prioritization is necessary to help enhance student pharmacists’ clinical competency upon graduation.

### 4.1. Program-Specific Tier Descriptions

Applying the concept of “understanding by design” to this context, the most logical consideration when prioritizing a topic is its prevalence within the scope of practice of the generalist pharmacist [9]. The lens we applied goes beyond prevalence of a topic or medical condition, and emphasizes the role of the pharmacist within that topic area. As pharmacist scope of practice evolves into independent prescribing of medications (e.g., self-administered hormonal contraception, smoking cessation therapy, self-care/minor ailment services), it makes sense that these areas be especially prioritized in the PharmD curriculum as pharmacists can expect to be providing these services immediately upon entry into practice. Similarly, topic areas that pharmacists directly manage through a collaborative practice framework and/or are frequently consulted by others should also be prioritized. When thinking about these topics, it is important to consider the full range of pharmacist practice areas to ensure the definitions are able to be applied as broadly as possibly. For example, consult requests from patients regarding minor ailment services may be more common in a community pharmacy setting, while requests to assist in streamlining antibiotic therapy may be more prevalent in an inpatient setting, and entry-level pharmacists must be prepared to manage both. Therefore, our description of prevalence within scope of practice of the generalist pharmacist attempts to address all of these practice areas by stratifying the degree of pharmacist involvement in the management and the frequency of consults within each topic area.

Another area that provides guidance is the prevalence of a topic in experiential education. As the experiential placements for each PharmD program are highly varied, it is important to take this into consideration when designing the pre-APPE curriculum. What types of practice settings and patient populations will students encounter during this highly formative portion of the curriculum? As an example, if all students are required to complete some or all of their experiential education within a specific health system, it makes sense to prioritize the medical conditions encountered there and emphasize topic areas within their specific pharmacist scope of practice to best prepare the students to be ready to apply their knowledge and skills in an authentic practice setting. Similarly, focusing on topics frequently encountered on national licensing exams can be used as another source of validation for a topic’s prioritization.

Lastly, the various Tiers were differentiated by the breadth and depth of information necessary to cover within the curriculum. This is one of the most valuable steps since it speaks on a more granular level than the prior factors, which focused on a topic’s presence or absence in various places (i.e., scope of practice, experiential setting, licensing exam preparation). When decisions need to be made about allocating time within the curriculum for both the foundational and clinical sciences, these are the factors that can inform those decisions. From the perspective of managing a specific condition, familiarity with clinical guidelines and goals of therapy are useful measures to apply—the higher priority topics simply require a stronger working knowledge of these items while topics of a lower priority more appropriately warrant awareness or ability to search for this information. It is important to emphasize that guidelines do change; some more often than others. Memorization of a guideline and/or goals of therapy will only serve students well in the short term, while more extensive training about how guidelines are made, how to critically evaluate the recommendations, and how to stay up to date with changes to the guidelines are also critical for assuring clinical competence. Therefore, although one component for the definition of each Tier 1 topic is “recall specific goals of therapy from memory”, it must be emphasized that additional coursework has been added to the curriculum in order to provide this more extensive training outside of the “pharmacotherapy curriculum”.

From the perspective of covering medication-specific information within the curriculum, the depth of content was based on place in therapy. Tier 1 topics require an emphasis on common first- and second-line agents, Tier 2A topics only require an emphasis on common first-line agents, and Tier 2B topics require a focus on names, clinical pearls, and major safety concerns. The Tier 1 and 2A distinctions focus on clinical management of the conditions, while the Tier 2B distinction was focused on individual medications for topics that may not otherwise be covered rather than clinical management of each topic. This was an important element to emphasize as medications that fall in this category may be those that are less commonly seen in practice or managed by pharmacists, but still come with significant risk of adverse effects, drug interactions, or toxicity that a pharmacist would be expected to know.

Although there is no significant role for most Tier 3 topics within the PharmD curriculum due to space constraints, these may be excellent topics that can be sprinkled into the curriculum, especially if the topic warrants a modification to the traditional management approach of a topic/condition within a higher Tier. For example, a student may not need to know how to actively manage or otherwise treat nephrolithiasis, but relevant clinical pearls for nephrolithiasis may be introduced briefly when deciding between medication options for another medical condition, and a history of nephrolithiasis may be the deciding factor for selecting one agent over another. In addition, there were a few Tier 3 topics that were deemed particularly pertinent to clinical practice in the Northwest region of the United States, which were allocated specific curricular time (e.g., physician-assisted dying, gender-affirming hormone therapy). Some Tier 3 topics will be incorporated into clinical cases as opportunities to apply core concepts and problem-solving during recitation.

Creating descriptions for the revised Tier structure was something the steering group originally tried to avoid doing, as there was concern that it would get faculty bogged down in too much detail and specificity. However, these descriptions have been extremely helpful for framing conversations about topic prioritization and allocating curricular time. Rather than focus on topic “importance”, these descriptions helped faculty keep their focus on identifying how much time and energy it would take to make a student proficient in a specific topic, how valuable that information will be to practice as an entry-level pharmacist, and whether or not that time is worthwhile relative to all the other curricular needs/demands. This was especially true for faculty who have a strong connection to a specific topic area and may have felt slighted by having their clinical area perceived as “less important”. Similarly, these definitions have also been helpful for faculty in the pharmaceutical sciences by providing application guidance for their content to prioritize the most relevant content for maximal learning, thereby enhancing clinical competence.

### 4.2. Prioritization and Categorization of Individual Topics

The process of recategorizing individual topics was also an extremely important step in this curricular design. Therefore, the comment period was essential for allowing faculty to identify topics that needed to be moved for a variety of reasons. For example, certain topics within the OB/GYN category were more appropriately covered in closer proximity to other topics (e.g., hypertensive disorders in pregnancy fit within the cardiovascular section alongside hypertension, gestational diabetes mellitus fit within the endocrine topics alongside type 1 and 2 diabetes mellitus). Similarly, other topics outside of the OB/GYN category had correlations to topics within the category (e.g., male hypogonadism, gender-affirming hormone therapy). Therefore, the OB/GYN category was broadened into a more inclusive “Sexual and Reproductive Health” category. Beyond identifying where topics are placed within the curriculum and how much time to dedicate to each, this schematic also informs the development of classroom exercises that intentionally integrate content from across the curriculum rather than operating within the silo of a specific disease state or topic area, especially if the focus is on helping assure clinical competence of students for generalist-pharmacist focus.

As previously mentioned, the memorization of drug facts and current guidelines will not assist in assuring clinical competence of current student pharmacists. Although the focus of this manuscript has been on prioritizing topics more traditionally characterized as the pharmacotherapy curriculum, it is important to note that additional elements have been implemented within our revised curriculum to support students’ development of their clinical competence. The redesigned, integrated, semester-based curriculum includes several course streams. These streams will support the scaffolding of topics across courses and longitudinally throughout the didactic curriculum. Course stream directors will be responsible for continually ensuring faculty adherence to the topic prioritization schematic when allocating content time, reviewing assessment items, and determining course policies each academic year.

### 4.3. Limitations

The major limitation of this work is the institution-specific nature of the end results (i.e., final Tier descriptions, final topic categorization and prioritization). Although the end results may differ by institution, our intention in presenting this work is to share the process and concepts utilized to inform our curricular topic prioritization. Because the curriculum has not officially launched, data evaluating the effectiveness of these methods are not yet available. However, each curriculum needs to start somewhere, and undergoing this process has helped provide a solid foundation with core philosophies that can be applied to future iterations when necessary.

### 4.4. Future Research

Several areas in need of further research have been identified during this curricular redesign process. Students will be provided with information about the curricular Tier levels and associated program-specific Tier descriptions. The team intends to evaluate whether providing this level of curricular information to students translates into enhancing their ability to better allocate study time and energy—and in turn, does this level of curricular focus help students become more APPE ready, practice ready, or even clinically competent?

The need for a corollary foundational science tier-system toolkit has also been identified, which might, perhaps, form the basis for national discussion and adoption. Furthermore, the intention to include some of the Tier 3 topics into the recitation courses, designed for integration and application of knowledge, provides an opportunity to study this approach as curriculum expanding without reducing the time allocated to priority core topics. The effectiveness of using Tier 3 topics as opportunities for foundational principle review and critical thinking also warrants research. Additionally, the role of elective coursework for covering niche practice areas and/or emerging therapy, and how to best incorporate this alongside a core-curriculum deserves investigation.

## 5. Conclusions

With ever-increasing demands on clinical competency, it is important to be intentional when determining curricular priorities. As pharmacists continue to increase their autonomy and scopes of practice within the healthcare arena, they are expected to acquire more knowledge, and there is the temptation to continually add to the curriculum without critically analyzing curricular content. Customizing a national framework to determine program-specific considerations for prioritizing topics within the pre-APPE curriculum can help faculty, and students alike, maintain focus on highly critical and foundational concepts, while also making sure not to completely disregard topics of lower priority. We have proposed such a framework for programs to utilize when facilitating conversations surrounding curricular reforms and topic prioritization and advocate for its use to inform competency-based curricular reform.

## Figures and Tables

**Table 1:** Tier Definitions within the ACCP Pharmacotherapy Didactic Curriculum Toolkit [10].

Tier	Definition	Topic Exemplars
Tier 1	Students receive education and training on this topic to prepare them to provide collaborative, patient-centered care upon graduation and licensure.	Hypertension Diabetes, types 1 and 2 Skin and soft tissue infections Nociceptive Pain
Tier 2	Students receive education and training on this topic, but additional knowledge or skills may be required after graduation (e.g., residency training or equivalent experience) to prepare them to provide collaborative, direct patient care.	Ventricular arrhythmias Pancreatitis Sickle cell disease Bone and joint infections Rheumatoid arthritis
Tier 3	Students and residents may not receive education and training on this topic; rather, they will be expected to obtain the required knowledge and skills on their own to provide collaborative, direct patient care if required in their practice.	Juvenile idiopathic arthritis Neurogenic bladder Polycystic kidney disease Autism spectrum disorders Cerebral palsy

**Table 2:** Program-Specific Tier Descriptions to Inform Curricular Decisions.

Tier	Pacific University School of Pharmacy Curriculum Prioritization Criteria
Tier 1A * & 1B *	Pharmacists are **actively managing** this topic/condition either **independently** or autonomously through a collaborative practice framework, and/or are **frequently consulted** on this topic/condition by patients or other members of the healthcare team. **Heavily emphasized** on **most** APPEs and NAPLEX Students are expected to perform the following actions/roles for each **Tier 1** topic prior to APPEs: ○ **Well-versed** in relevant guideline(s) for treatment & management of condition ○ **Recall specific** goals of therapy from memory (if applicable) ○ Describe place in therapy, mechanism of action, kinetics (if applicable) general dosing/dosing formulations, safety and monitoring, and clinically relevant side effects of common **first AND second line agents**
Tier 2A	Pharmacists are **contributing to** the management of this topic/condition autonomously through a collaborative practice framework in a **few specialized areas of practice** and/or are **occasionally consulted** by patients or other members of the healthcare team on this topic/condition. **Moderately emphasized** on **some** APPEs and NAPLEX Students are expected to perform the following actions/roles for each **Tier 2A** topic prior to APPEs: ○ **Aware** of the relevant guideline(s) for treatment & management of condition ○ **Recall general** goals of therapy from memory (if applicable) ○ Describe place in therapy, mechanism of action, kinetics (if applicable), general dosing/dosing formulations, safety and monitoring, and clinically relevant side effects of common **first** line agents only
Tier 2B	Pharmacists are **contributing** to the management of this topic/condition autonomously through a collaborative practice framework in **very specialized areas of practice** and/or are **rarely consulted** by patients or other members of the healthcare team on this topic/condition. **Occasionally appears** on APPEs and NAPLEX Students are expected to perform the following actions/roles for each **Tier 2B** topic prior to APPEs: ○ **Can search for** the relevant guideline(s) for treatment & management of condition ○ **Can search for** goals of therapy (if applicable) ○ Recall name of **common first line agents** and/or **clinical pearls for unique agents**/**scenarios** (only if relevant) and **identify major safety concerns**
Tier 3 †	Pharmacists are **not typically involved** in the management of this topic/condition and/or are **hardly ever consulted** by patients or other. Members of the healthcare team on this topic/condition **Rarely appears** on APPEs and NAPLEX Condition is generally not treated with pharmacotherapy agents Students may hear about the topic or be introduced to very surface-level information as time allows

* Tier 1A and 1B topics both have the same SoP criteria (listed above). The only difference may be that Tier 1A topics would take highest priority in any situation where curricular decisions are made (if applicable). † Certain items designated as Tier 3 by ACCP were considered pertinent to pharmacy practice in the Northwest United States region, and therefore reprioritized into a higher tier. Abbreviations: APPEs: Advanced Pharmacy Practice Experiences; NAPLEX: North American Pharmacist Licensure Exam.

## Data Availability

Not applicable.
